# Experiences and perceptions of health professionals towards the quality of care for people living with HIV in Tunisia: a qualitative study

**DOI:** 10.11604/pamj.2023.46.4.35184

**Published:** 2023-09-06

**Authors:** Mariem Zribi, Nadia Ben Mansour, Hayet Moussa, Hichem Ben Hassine, Hajer Aounallah-Skhiri

**Affiliations:** 1 National Institute of Health, Tunis, Tunisia,; 2 Faculty of Medicine of Tunis, University of Tunis El Manar, Tunis, Tunisia,; 3 Higher Institute of Human Sciences of Tunis, Tunis, Tunisia,; 4 Science Shop, Pasteur Institute of Tunis, Tunis, Tunisia

**Keywords:** HIV, health care professionals, perspective, quality of health care, attitude

## Abstract

**Introduction:**

human immunodeficiency virus (HIV) infection continues to be a public health issue, especially in low- and middle-income countries, including Tunisia. In 2020, 32% of people living with HIV were on treatment. Management of HIV infection remains a real challenge for both patients and care providers. Our study aimed to describe the perceptions and attitudes of health professionals toward care for people living with HIV (PLHIV).

**Methods:**

a qualitative study was carried out between 2020 and 2021. Interviews with key informants were conducted in order to identify the strengths and weaknesses of the care for PLHIV, as well as their suggestions for improvement. Eight key informants in HIV care from different profiles were interviewed: healthcare providers from a specialized university hospital, the Ministry of Health, and civil society representatives working in the field of HIV. Interviews were transcribed and analyzed thematically using “QDA Miner” software.

**Results:**

the frequency of PLHIV who have interrupted medical follow-up was perceived as worrisome, and increasing. Along with individual factors, non-adherence to treatment was also attributed to systemic factors related to stock shortages, geographical inaccessibility, and shortfall in human resources. Stigmatization of PLHIV in healthcare facilities outside the specialized hospital was also highlighted. This has been linked to gaps in the training of care providers on the modes of the virus transmission.

**Conclusion:**

health professionals face many challenges in the care of PLHIV. There is an urgent need to improve treatment availability and accessibility, strengthen social assistance for PLHIV and fight against stigmatization, especially in healthcare settings.

## Introduction

Since the 1980s, when its first cases were described, HIV infection continues to be a public health problem in many parts of the world [1,2]. According to reports from the Joint United Nations Program on HIV/AIDS, 75.7 million people have been affected and there have been 32.7 million deaths from AIDS-related diseases since the beginning of the epidemic. In 2020, the Joint United Nations Programme on HIV and AIDS (UNAIDS) estimated that there were 38 million people living with HIV (PLHIV) worldwide [3]. Recent advances in biological sciences and clinical research have successfully improved screening, virological monitoring, and treatment efficiency and security. All these factors have considerably improved the survival and quality of life for PLHIV, in which anti-retroviral therapy (ART) had played an important role [4]. In fact, countries where ART has been made nationally available have shown the highest reduction in HIV-related morbidity and mortality [5]. Nevertheless, high levels of adherence to ART (at least 95%) are needed to keep an undetectable viral load and to ensure optimal benefits for better clinical outcomes not only to the patient himself but also to prevent transmission to negative HIV sexual partners [6,7].

Despite the historic turning point induced by ART in HIV infection, the UNAIDS goal of 90% viral suppression has not been reached, partly because of poor adherence [3]. In 2020, 27% of PLHIV worldwide still do not have access to antiretroviral treatment [3]. Tunisia remains away from achieving HIV management goals compared to other countries. The Joint United Nations Programme on HIV and AIDS(UNAIDS) estimates a 61% increase in new cases in 2020 compared to 2010 and a 45% increase in AIDS-related deaths compared to 2010. Only 32% of PLHIV have access to antiretroviral treatment [8]. This is despite the fact that Tunisia joined the Political Declaration on HIV/AIDS “Intensifying our efforts to eliminate HIV/AIDS” in June 2011 in New York [9]. These facts are consistent with a cry of distress and protest sent by non-governmental organizations (NGO) dealing with PLHIV, deploring the emergence of drug resistance and deterioration of health status [10]. It should be emphasized that several reports have been published about HIV/AIDS care, but none have been published on implementing interventions to enhance PLHIV adherence. Original research on quality of healthcare in Tunisia, and in particular on the emergent HIV infection, is scarce.

Within the framework of the call launched by Science Shop “Science together” to express social needs, the Tunisian Association of Positive Prevention ATP+ (*Association Tunisienne de Prévention Positive*), an association involved in promoting better care for PLHIV, submitted the request to communicate experiences of PLHIV in health care settings. ATP+ had “co-developed” the project with researchers of different scientific backgrounds (medical, epidemiologist, and social science). An original research design was adopted based on the reformulation exercise of the ATP+ request, in order to inform about factors behind treatment non-adherence among PLHIV. The overall aim was to inform future intervention designs applicable at the health structure level. The study took the design of qualitative original research consisting of interviewing PLHIV and stakeholders of different perspectives, in order to assure the comprehensiveness of the assessment, as well as the applicability of the recommendations. This paper reports the key informants (KI) study, aiming to study perceptions, attitudes and experiences of healthcare professionals of different profiles in relation to care for PLHIV.

## Methods

**Study design:** this is a qualitative study carried out over a total period of nine months between 2020 and 2021.

**Study population and data collection:** health and social care providers (HCP) sampling was purposive based on critical case sampling. We included six health and social care providers (HCP) working in the hospital with different profiles (Table 1): specialist (1), nurses (2), psychologist (1), worker (1), and pharmacist (1). Expert sampling will be used for other stakeholders from the Ministry of Health (1), and civil society representatives (1) in the field of HIV care.

**Table 1:** interviewees’ profiles

Participant’s profile	Number
Specialist (physician)	1
Nurse	2
Worker	1
Psychologist	1
Pharmacist	1
Representative of Health Ministry working in the field of HIV	1
Representative of civil society working in the field of HIV	1

**Data collection:** interviews were carried out using a semi-structured guide administrated by two interviewers, covering topics as follows: health care providers and NGOs stakeholders: experience with HIV treatment adherence, expectations and suggestions to improve treatment adherence. Ministry of Health representative: supply of treatment, perception about patients´ adherence, and implemented measures regarding prevention and treatment.

**Data analysis:** all interviews were transcribed and thematically analyzed using “QDA Miner” software [11]. Our coding strategy has been described by Miles M. and Huberman [12], as halfway between the two “preconceived and inductive” approaches. Two research team members independently reviewed transcripts and developed a consensus plan to identify recurring themes and variants within the framework of qualitative content analysis (Corbin & Strauss, 2014). They independently designed a coding scheme that categorizes segments of data by topic rather than content (words, sentences...), and variants were assigned to each pre-defined code within the framework of qualitative content analysis (Corbin & Strauss, 2014) [13]. Participants´ suggestions coding proceeded inductively, allowing for new main categories to emerge. The general coding scheme or the major categories in which codes were deductively designed, has been defined according to the basic elements of quality of care (continuity and comprehensiveness of care, accessibility, empathy, effectiveness, and dignity) and suggestions for improvement.

**Ethical considerations:** the biomedical ethical committee of the Pasteur Institute of Tunis has approved this research project. In addition, it approved all revisions and amendments to the research protocol, the information letter, and the consent form.

## Results

Quotes on major quality care attributes and suggestions for improvement are summarized in **Table 2**. The most striking issues raised during the individual interviews were:

**Table 2:** quotes on major quality care attributes and suggestions for improvement

Quality of care attributes	Quotes	Suggestions for improvement
Continuity of care	“Interruption of treatment was mainly observed among drug addicts, homeless people who interrupt their medical follow-up”.	“It is crucial to establish free care related to opportunistic infections which are the cause of the patient's death”.
	“Those who accept the disease continue their follow-up” ... “They are young patients, with good awareness”.	
Access to antiretroviral treatment	“Stock shortage is mainly due to lack at the central level, lack of treatment for children (syrup form) is also frequent”.	“Pressure must be made in the central pharmacy and towards decision-makers to ensure availability of treatment”.
	“In case of need we “troubleshoot” the patients while waiting to have their treatment, we do everything to manage and provide the treatment to our patients if some medicines are lacking”.	
	“This was due to the difficulties in ART management and prescription”.	
Coordination between healthcare providers	“The medical, psychosocial, and social management is made in parallel in the department”.	“Assigning a social worker to serve permanently in the department is crucial”.
	“Therapeutic education is coordinated between the doctor, the psychologist, and the social assistant, but it is not enough”.	“The role of the social assistant would be to coordinate with the Ministry of Social Affairs for comprehensive care of the PLHIV”.
	“The social assistant is not always available. Lack of paramedical staff is also noticeable”.	“Strengthening human resources underway (social workers and psychologists) and coordinating with the Ministry of Social Affairs for a comprehensive management of PLHIV is essential”.
Stigmatization	“Stigmatization is due to a culture: this disease is considered taboo and PLVIH are rejected”.	“We should improve awareness and education of health care personnel on modes of contamination”.
	“Unlike our department where PLHIV are “spoiled”, our patients are exposed to discrimination and stigmatization in other departments such as the maternity department, stomatology, ophthalmology, and stomatology consultations”.	“The provider’s job is not only to prescribe but also to ensure that the message has been received by the patient (information-feedback), to have an empathic relationship with the patient”.
	“They feel rejected, they feel bad”.	

**Continuity and comprehensiveness of care for people living with HIV:** the majority of professionals interviewed rated the incidence of PLHIV who interrupted their medical follow-up as increasing *“, especially after the 2011 events”*. This was related mainly to social and economic difficulties. Some vulnerable groups were perceived as more at risk than others. *“Interruption of treatment was mainly observed among drug addicts, homeless people who interrupt their medical follow-up”, “those with substance abuse or psychosocial difficulties are particularly susceptible”*. In contrast, PLHIV who are likely to continue their medical follow-up was judged as younger, more educated and with positive attitudes. *“Those who accept the disease continue their follow-up”. “They are young patients, with good awareness”, “smart, well-educated and observant generation”*.

**Access to antiretroviral treatment:** in addition to individual factors *“lack of financial resources and association with other diseases (such viral hepatitis C)”*, antiretroviral therapy (ART) stock-outs were a recurrent problem and perceived as the key factor of non-adherence. *“It causes serious resistance, there are patients who interrupt their treatment for three years because of repetitive stock breaks”, “Stock shortage is mainly due to lack at the central level, lack of treatment for children (syrup form) is also frequent”*. Nevertheless, some adjustment measures have been highlighted. Generally, there is a reserve stock at the local level of the internal pharmacy of the department *“In case of need we “troubleshoot” the patients while waiting to have their treatment, we do everything to manage and provide the treatment to our patients if some molecules are lacking”, and also at the central level. “In 2020 we are in the process of restoring and correcting while having a security stock, especially in the current circumstances related to COVID-19”*. Moreover, the National HIV Control Program was considered a prerequisite for universal access to anti-retroviral treatment for people who need it in Tunisia *“all known PLHIV, (including illegal immigrants) are on treatment”*.

Although no conditions of restriction on access to anti-retroviral treatment were noted, there were still questions regarding the attainment of all eligible persons, especially in the COVID-19 epidemic context. *“So, for the first 90, we´re still very far from the target. We are currently undertaking mass screening programs, but the COVID-19 epidemic is affecting the population adherence”*. Geographic inaccessibility was stressed by the majority of the interviewees as a structural and persistent barrier, well in advance of the outbreak. In fact, treatment is only provided through four care sites (Tunis-Sfax-Monastir-Sousse). *“Patients have transport difficulties to access specialized centers in HIV to get adequate therapy and follow up”*. Exceptional during the lockdown period, a form of decentralization of the ART dispensation was established in collaboration between the Ministry of Health and NGOs working in the field of HIV. *“We tried to improve the treatment accessibility during the COVID-19 epidemic by establishing a form of decentralization of the ART. NGO ensured the recovery of treatments, transport, and psychological and social assistance. We aimed essentially to reduce the exposition of patients”*. However, the usual dispensation of anti-retroviral therapy being carried out in four referral hospitals, the decentralization of ART delivery is not envisaged currently. The main reasons were the limited number of patients´ beneficiaries, and the complexity of medication management and prescription that requires some expertise. *“This was due to the difficulties in ART management and prescription”*.

**Other dimensions of people living with HIV care: Empathy, coordination and dignity:** the importance of establishing a good patient-health professional relationship was highly recognized during the interviews. *“Patients tell us about their psychosocial, emotional and illness-related problems. We listen to our patients.” “The provider´s job is not only to prescribe, but also to ensure that the message has been received by the patient (information-feedback), to have an empathic relationship with the patient”*. All the same, the coordination between the various actors in therapeutic education was perceived positively by healthcare providers. *“The medical and psychosocial management is made in parallel in the department”*. However, therapeutic education was deemed insufficient by the NGO representative. *“Therapeutic education is coordinated between the doctor, the psychologist and the social assistant, but it is not enough”*. This can be explained by the lack of human resources, some profiles have been specifically mentioned by professionals *“The social assistant is not always available (not assigned specifically to this department). Lack of paramedical staff is also noticeable”*. Moreover, overall coordination with other departments has also been criticized. *“When I first received PLHIV, the radiology appointments are far away. Serologies drag on and take time to be ready. All this is a waste of time for the patient.”* Finally, respect for the dignity of PLHIV (one of the fundamental rights of patients in the general care setting) was positively assessed within the unit where the professionals interviewed practiced: *“PLHIV are treated like other patients”*. Indeed, the medical and paramedical environment are accustomed to the care for PLHIV. *“We maintain a relationship of trust and respect with PLHIV.”*

**Stigmatization of people living with HIV:** the interviewed professionals particularly emphasized stigma and discrimination experienced by PLHIV in other care-giving contexts. *“There is a stigma of PLHIV in other departments, especially outside our hospital”. “Unlike our department where PLHIV are “spoiled”, our patients are exposed to discrimination and stigmatization in other departments such as the maternity department, ophthalmology and stomatology consultations”*. The majority of professionals interviewed reported these phenomena of stigma of PLHIV to a problem of training and awareness among care providers: *“Discrimination is present in every country in the world; an Iranian study has shown that the cause is a lack of knowledge about how HIV is transmitted. All the same, a knowledge gap on HIV transmission patterns is noticeable among health staff in Tunisian hospitals”*. Moreover, cultural factors were also reported: *“Stigmatization is due to a mentality: this disease is considered taboo and PLVIH are rejected”*.

**Suggestions for improving the care for people living with HIV:** a set of suggestions were made by the various stakeholders to improve HIV management. The first set was related to the microlevel (care setting), as education and training of health professionals. *“We should improve awareness and education of health care personnel on modes of contamination”*. Moreover, enhance the coordination between different stakeholders and caregivers were deemed very important, practical measures were suggested such as: designating a focal point for PLVIH care in other departments (Radiology, biological analysis). *“Assigning a social assistant to serve permanently in the department is crucial”. “The role of the social assistant would be to coordinate with the Ministry of Social Affairs for comprehensive care of the PLHIV”. “Strengthening human resources underway (workers and psychologists) and coordinating with the Ministry of Social Affairs for a comprehensive management of PLHIV is essential”*. On a macro level participant called to improve treatment availability: *“Pressure must be made in the central pharmacy and towards decision-makers to ensure availability of treatment”*. Finally, provide opportunistic infections free of charge care for PLHIV was deemed essential to reduce mortality among PLHIV. *“It is crucial to establish free care related to opportunistic infections which are the cause of the patient's death”*.

## Discussion

HIV research in Tunisia is far below what is needed, and diverse areas of HIV management research are still not covered adequately and robustly [14]. This study reports on an original research design based on reformulation of the civil society need, in order to inform future interventions to improve HIV management in Tunisia. Using a qualitative approach, we aimed to study the perceptions and experiences of healthcare professionals in relation to the care for PLHIV. The positive aspects identified were mainly the awareness of the importance of empathy, listening, and trust in the medical management of patients. Moreover, the commitment to universal access to antiretroviral treatment for known PLHIV in Tunisia was formalized as a priority. The resilience of the treatment delivery during the COVID-19 epidemic was also noticeable. However, negative points were also highlighted, the frequency of PLHIV who have interrupted their medical follow-up and treatment was perceived as increasing, and some vulnerable groups were reported to be most exposed (drug use, socio-economic precariousness, isolation, etc.), Furthermore, factors related to the health system aggravate the problem of therapeutic non-compliance, such as stock shortages and geographical inaccessibility, stigmatization of PLHIV in departments that are not accustomed to HIV management. Fear of being infected, rejection, and blame were considered as the direct causes of stigmatization in a care setting. The unavailability of ART was reported by healthcare providers as a barrier for PLHIV to access treatment, especially concerning some molecules and forms (for example, the syrup form for children). These results are corroborated by several studies in other countries, which showed that poor adherence to therapy delayed management and consequently worsened the virus infection [15,16].

Apart from pharmacological therapy, the second pillar of good HIV care for patients is interdisciplinary management that takes into account therapeutic education, psychological support, and social and economic assistance [17]. In our study, coordination between different stakeholders was perceived as insufficient. This was related to the lack of numbers of healthcare providers from some profiles (hospital workers and social assistants mainly). Support for PLHIV involves managing HIV diagnosis announcement, psychological support, therapeutic education, nutritional education, and economic or legal assistance [18]. To achieve this holistic management, overall coordination between different departments and specialties is fundamental. This aspect was criticized according to our results and needs to be improved.

Stigma is central to the problems faced by patients [19]. This attitude was widely reported in the healthcare setting [20,21]. Stigma and discrimination hamper the efficient care provided to PLHIV by limiting access to preventive services [22], engagement in care, and adherence to antiretroviral treatment [23,24]. Stigma and discrimination still represent the main barriers to accessing screening and HIV care. In 2018, about 65% of women aged 15 to 49 reported discriminatory attitudes towards HIV in Tunisia [25]. The different aspects of stigma in our assessment were manifested by avoiding contact with the patient, the patient passing the last to consultations, and an unpleasant behavior while benefiting from an act of care (stomatology, ophthalmology, maternity departments...). “The patient waits to be the last one to pass to consultations”. Stigma within these care settings was explained by fear of contamination by healthcare professionals or by lack of awareness and ignorance of ways of contamination by the virus.

Similarly, a study conducted at a hospital in Ghana showed that nurses postponed care for PLHIV remotely to avoid contact with them [26]. In addition, a recent Tunisian study has reported that some doctors are reluctant to apply invasive methods to these patients because they fear contamination [27]. This is in contrast with the legislation in force mentioning that everyone living with HIV in Tunisia has the right to dignity, respect, privacy and supportive care that meets their physical and psychological needs [27]. We believe that the part of discussion about ways to improve the PLHIV care was very informative for improving the dimensions of HIV management. In light of our results, we emphasize the importance of promoting the Human Rights Model of Care [27], ensuring ongoing training of health personnel on modes of contamination as well as ethical aspects of management [17] (confidentiality, anti-discrimination and stigma of PLHIV), improving ART availability [15] and strengthening multidisciplinary management based on medical management, psychological support and social and economic assistance [24].

The strengths of our study came from being guided by a social need. As a result of this work, training videos on HIV care based on human rights were produced. These 2D animations were made in order to increase the level of awareness among care providers. They included a video on “The right to privacy”, a video on “the right to non-stigmatization and non-discrimination”, and a video on “HIV transmission patterns” [28].

**Limitations:** the interpretation of our results should consider the methodological limitations of the qualitative study. The latter are mainly related to the sample studied, which limits the generalization of the results. Furthermore, the social desirability bias represents an issue in our work, since key informants were partially questioned about their own practices. Therefore, in a second phase, we aim to validate our results by their confrontation with PLHIV interviews.

## Conclusion

This study highlighted challenges faced by health professionals and stakeholders in HIV care. Some aspects of HIV care were assessed positively, such as the comprehensiveness of care in specialized departments, as well as the resilience of the treatment delivery during the COVID-19 epidemic. However, there is an urgent need to improve the treatment availability and accessibility, strengthen social and medical assistance for PLHIV, and fight against stigmatization, especially in healthcare settings.

### What is known about this topic

- *Human immunodeficiency virus (HIV) infection continues to be a major public health issue, especially in low- and middle- income countries, including Tunisia;*
- *Adherence to HIV care and follow-up remains a real challenge for both patients and care providers*.

### What this study adds

- *To the best of our knowledge, the present study is the first to describe the experiences and perceptions of health professionals toward quality of care for people living with HIV in Tunisia;*
- *This study provides benchmark data to use by health authorities in order to improve the quality of care for people living with HIV*.
